# Supplementary material for: Augmented cartilage regeneration by implantation of cellular versus acellular implants after bone marrow stimulation: a systematic review and meta-analysis of animal studies
Source: PeerJ. 2017 Oct 27;5:e3927. doi: 10.7717/peerj.3927 (PMC5661456; doi:10.7717/peerj.3927)
Supplement: Supplemental Information 1 [file peerj-05-3927-s001.pdf]

Augmented cartilage regeneration by implantation of cellular versus acellular implants after bone marrow stimulation: a systematic review and meta-analysis of animal studies

by Pot *et al.*

Rationale for conducting the meta-analysis

For patients, localized cartilage defects can have detrimental long term effects such as joint dysfunction, pain, and degenerative osteoarthritis. Upon cartilage damage, the avascular nature of cartilage prevents spontaneous healing [1]. Clinical treatments, including bone marrow stimulation techniques (microfracturing/subchondral drilling), result in temporary clinical improvement. Therefore, the demand for improved cartilage regeneration persists [2].

Cartilage regeneration may be improved by tissue engineering and regenerative medicine (TERM) in addition to bone marrow stimulating techniques. For clinical application of new regenerative medicine and tissue engineering strategies, the effectiveness of implants, biologics and cells needs to be proven. The aim of this systematic review and meta-analysis is to assess all current evidence for the efficacy of articular cartilage regeneration using cellular implants versus acellular implants.

Contribution of this meta-analysis in the light of published related reports

In a previous systematic review and meta-analysis on animal models, we investigated acellular implants (vs. bone marrow stimulation), specifically focusing on material properties of implants and the addition of biologics [3]. Implantation of acellular biomaterials in addition to bone marrow stimulation was more effective in the regeneration of cartilage *in vivo* than bone marrow stimulation alone, which was further improved by use of biologics.

When biomaterials are loaded with cells, bone marrow stimulation may be even more effective. The aim of this systematic review and meta-analysis is to assess all current evidence for the efficacy of articular cartilage regeneration using cellular and acellular implants and more specifically loading of implants with (1) stem cells versus somatic (differentiated) cells, (2) different cell types (*e.g.* chondrocytes, MSCs and ADSCs), and (3) culture conditions of cells (*e.g.* direct use after harvesting, *in vitro* expansion and/or differentiation). No other systematic review and meta-analysis has been performed to assess the effect of the loading of cells to implants on cartilage regeneration in animal models.

References

[1] Buckwalter JA, Saltzman C, Brown T. The impact of osteoarthritis: implications for research.

Clin Orthop Relat Res 2004; 427 Suppl: S6-15

[2] Saris D, Price A, Widuchowski W, Bertrand-Marchand M, Caron J, Drogset JO, et al. Matrix-applied characterized autologous cultured chondrocytes versus microfracture: two-year follow-up of a prospective randomized trial. Am J Sports Med 2014; 42: 1384-1394.

[3] Pot MW, Gonzales VK, Buma P, IntHout J, van Kuppevelt TH, de Vries RBM, et al. Improved cartilage regeneration by implantation of acellular biomaterials after bone marrow stimulation: a systematic review and meta-analysis of animal studies. PeerJ 2016; 4: e2243-2269.
