# Supplementary figures and images for: Augmented cartilage regeneration by implantation of cellular versus acellular implants after bone marrow stimulation: a systematic review and meta-analysis of animal studies
Source: PeerJ. 2017 Oct 27;5:e3927. doi: 10.7717/peerj.3927 (PMC5661456; doi:10.7717/peerj.3927)

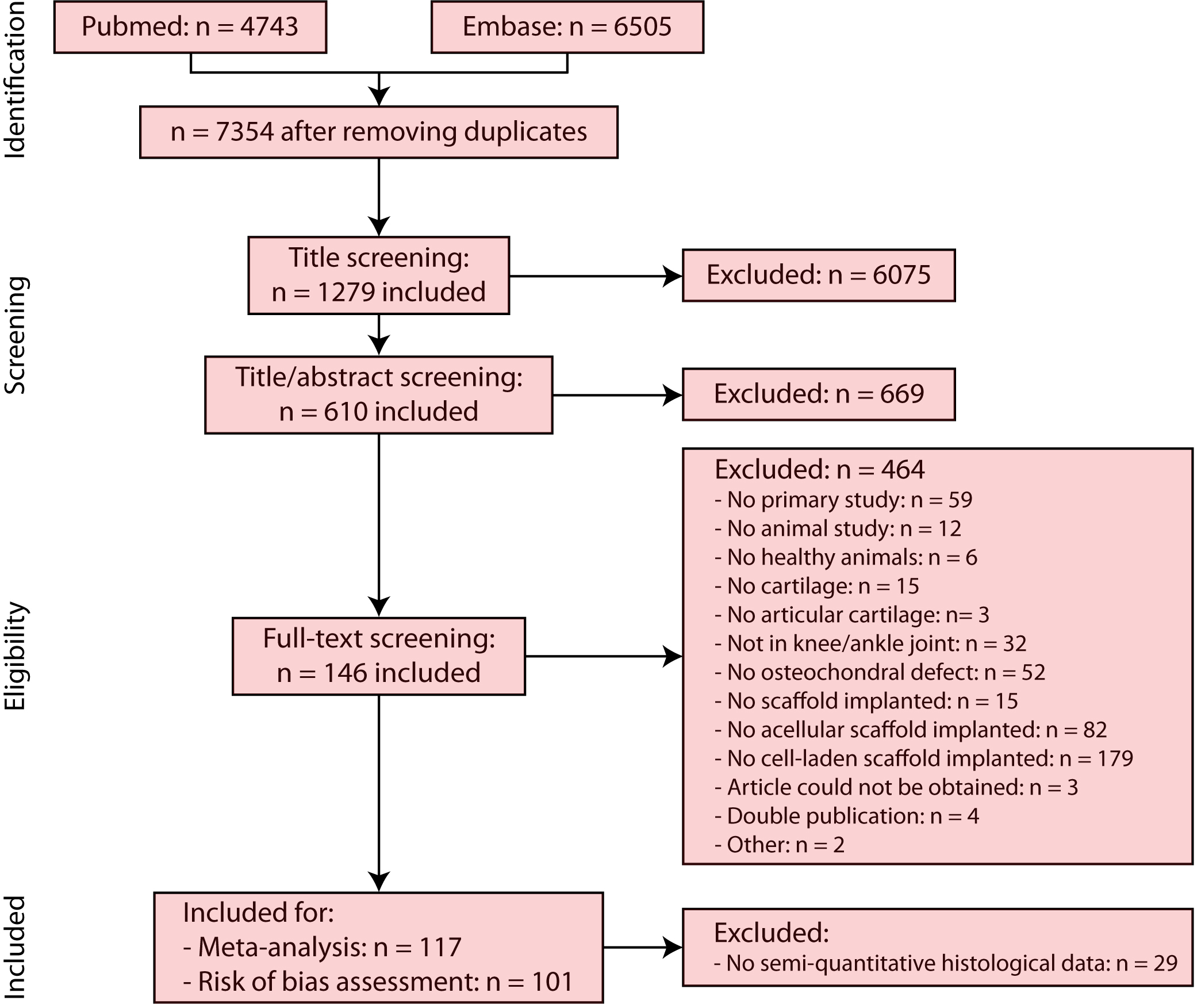

Supplement: Supplemental Information 2 [file peerj-05-3927-s002.jpg]
