# Supplementary material for: Augmented cartilage regeneration by implantation of cellular versus acellular implants after bone marrow stimulation: a systematic review and meta-analysis of animal studies
Source: PeerJ. 2017 Oct 27;5:e3927. doi: 10.7717/peerj.3927 (PMC5661456; doi:10.7717/peerj.3927)
Supplement: Supplemental Information 4 [file peerj-05-3927-s004.pdf]

Supplementary Information 2. Exclusion criteria.

Title screening

1. No primary study
2. No animal study
3. No bone marrow stimulation performed
4. Osteoarthritis models
5. Ex vivo studies
6. Deceased animals

Title/abstract screening

1. No primary study
2. No animal study
3. No bone marrow stimulation performed
4. Osteoarthritis models
5. Ex vivo studies
6. Deceased animals

Full-text screening

1. No primary study
2. No animal study
3. No ‘healthy’ animals (*i.e.* arthritis models)
4. No cartilage (*i.e.* bone or other tissue)
5. No articular cartilage (*i.e* elastic cartilage)
6. Not in the knee or ankle joint (*i.e.* ear, rib)
7. No bone marrow stimulation performed
8. No scaffold implanted
9. No cell-free scaffold implanted
10. No cell-laden scaffold implanted
