# Supplementary material for: Augmented cartilage regeneration by implantation of cellular versus acellular implants after bone marrow stimulation: a systematic review and meta-analysis of animal studies
Source: PeerJ. 2017 Oct 27;5:e3927. doi: 10.7717/peerj.3927 (PMC5661456; doi:10.7717/peerj.3927)
Supplement: Supplemental Information 6 [file peerj-05-3927-s006.pdf]

Supplementary Information 4. Study characteristics of included studies: abbreviations and references

Abbreviations

Biomaterial
CDBM: cancellous demineralized bone matrix
DCBM: decellularized cancellous bone matrix
DCM: decellularized cartilage matrix
GMP: gelatin microspheres
ICP: Injectable calcium phosphate
KLD hydrogel: self-assembling hydrogel from peptide sequences $(RADA)_4$ and $(KLDL)_3$
OPF: oligo(poly(ethylene glycol) fumarate)
PCL: polycaprolactone
PEG: poly(ethylene glycol)
PEOT/PBT: poly(ethylene oxide terephthalate)/poly(butylene terephthalate)
PGA: poly(glycolic acid)
PHBV: (poly(3-hydroxybutyric acid-co-3-hydroxyvaleric acid)
PLA: poly(lactic acid)
PLCL: poly(L-lactide-co-caprolactone)
PLGA: poly(lactic-co-glycolic acid)
PLLA: poly-l-lactide acid)
PVA: poly(vinyl alcohol)
TCP: tricalcium phosphate
TMC: N,N,N-trimethylchitosanchloride

Cells
ACI: autologous chondrocyte implantation
ASCs: adipose-derived stem cells
BMAC: bone marrow aspiration concentrate
BM-MNCs: bone marrow-derived mononuclear cells
EPCs: endothelial progenitor cells
hUCB-MSCs: human umbilical cord blood-derived mesenchymal stem cells
MPCs: bone marrow-derived progenitor cells
MSCs: mesenchymal stem cells
SDMSCs: synovium derived mesenchymal stem cells
SVF: stromal vascular fraction

Biologicals
bBMPs: bovine bone morphogenetic proteins
BMP: bone morphogenetic protein
CBD-BMP: Collagen binding domain bone morphogenetic protein
EPO: erythropoietin
FGF: fibroblast growth factor
IGF: insulin-like growth factor
PRP: platelet-rich plasma
rhAC: recombinant acid ceramidase
rhBMP: recombinant human bone morphogenetic protein
TGF-β: transforming growth factor-β

Other
ECM: extracellular matrix

References

1. Ahn JH, Lee TH, Oh JS, Kim SY, Kim HJ, Park IK, et al. Novel hyaluronate-atelocollagen/beta-TCP-hydroxyapatite biphasic scaffold for the repair of osteochondral defects in rabbits. Tissue Engineering Part A 2009; 15: 2595-2604.
2. Albrecht F, Roessner A, Zimmermann E. Closure of osteochondral lesions using chrondral fragments and fibrin adhesive. Archives of Orthopaedic and Trauma Surgery 1983; 101: 213-217.
3. Araki S, Imai S, Ishigaki H, Mimura T, Nishizawa K, Ueba H, et al. Improved quality of cartilage repair by bone marrow mesenchymal stem cells for treatment of an osteochondral defect in a cynomolgus macaque model. Acta Orthopaedica 2015; 86: 119-126.
4. Bai T, Shu J, Wang J, Lu J, Li W, Pu B. Experimental research on repair of rabbit articular cartilage deffects with composite of autologous cell-carriers. Chinese Journal of Reparative and Reconstructive Surgery 2008; 22: 487-491.
5. Bal BS, Rahaman MN, Jayabalan P, Kuroki K, Cockrell MK, Yao JQ, et al. In vivo outcomes of tissue-engineered osteochondral grafts. Journal of Biomedical Materials Research Part B 2010; 93: 164-174.
6. Barron V, Merghani K, Shaw G, Coleman CM, Hayes JS, Ansboro S, et al. Evaluation of Cartilage Repair by Mesenchymal Stem Cells Seeded on a PEOT/PBT Scaffold in an Osteochondral Defect. Annals of Biomedical Engineering 2015.
7. Barron V, Neary M, Mohamed KM, Ansboro S, Shaw G, O'Malley G, et al. Evaluation of the Early In Vivo Response of a Functionally Graded Macroporous Scaffold in an Osteochondral Defect in a Rabbit Model. Ann Biomed Eng 2016; 44: 1832-1844.
8. Betsch M, Schneppendahl J, Thuns S, Herten M, Sager M, Jungbluth P, et al. Bone Marrow Aspiration Concentrate and Platelet Rich Plasma for Osteochondral Repair in a Porcine Osteochondral Defect Model. PLoS One 2013; 8.
9. Betsch M, Thelen S, Santak L, Herten M, Jungbluth P, Miersch D, et al. The role of erythropoietin and bone marrow concentrate in the treatment of osteochondral defects in mini-pigs. PLoS One 2014; 9: e92766.
10. Caminal M, Peris D, Fonseca C, Barrachina J, Codina D, Rabanal RM, et al. Cartilage resurfacing potential of PLGA scaffolds loaded with autologous cells from cartilage, fat, and bone marrow in an ovine model of osteochondral focal defect. Cytotechnology 2015.
11. Cao Z, Hou S, Sun D, Wang X, Tang J. Osteochondral regeneration by a bilayered construct in a cell-free or cell-based approach. Biotechnology Letters 2012; 34: 1151-1157.
12. Chang CH, Kuo TF, Lin FH, Wang JH, Hsu YM, Huang HT, et al. Tissue engineering-based cartilage repair with mesenchymal stem cells in a porcine model. Journal of Orthopaedic Research 2011; 29: 1874-1880.
13. Chang NJ, Lam CF, Lin CC, Chen WL, Li CF, Lin YT, et al. Transplantation of autologous endothelial progenitor cells in porous PLGA scaffolds create a microenvironment for the regeneration of hyaline cartilage in rabbits. Osteoarthritis Cartilage 2013; 21: 1613-1622.
14. Chen WC, Yao CL, Wei YH, Chu IM. Evaluating osteochondral defect repair potential of autologous rabbit bone marrow cells on type II collagen scaffold. Cytotechnology 2011; 63: 13-23.
15. Chen J, Chen H, Li P, Diao H, Zhu S, Dong L, et al. Simultaneous regeneration of articular cartilage and subchondral bone in vivo using MSCs induced by a spatially controlled gene delivery system in bilayered integrated scaffolds. Biomaterials 2011; 32: 4793-4805.

16. Chen Z, Zhao M, Liu K, Wan Y, Li X, Feng G. Novel chitosan hydrogel formed by ethylene glycol chitosan, 1,6-diisocyanatohexan and polyethylene glycol-400 for tissue engineering scaffold: in vitro and in vivo evaluation. Journal of Materials Science: Materials in Medicine 2014; 25: 1903-1913.
17. Cheng A, Kapacee Z, Peng J, Lu S, Lucas RJ, Hardingham TE, et al. Cartilage repair using human embryonic stem cell-derived chondroprogenitors. Stem Cells Translational Medicine 2014; 3: 1287-1294.
18. Christensen BB, Foldager CB, Hansen OM, Kristiansen AA, Le DQ, Nielsen AD, et al. A novel nano-structured porous polycaprolactone scaffold improves hyaline cartilage repair in a rabbit model compared to a collagen type I/III scaffold: in vitro and in vivo studies. Knee Surgery, Sports Traumatology, Arthroscopy 2012; 20: 1192-1204.
19. Chung JY, Song M, Ha CW, Kim JA, Lee CH, Park YB. Comparison of articular cartilage repair with different hydrogel-human umbilical cord blood-derived mesenchymal stem cell composites in a rat model. Stem Cells Translational Medicine 2014; 5.
20. Cohen SB, Meirisch CM, Wilson HA, Diduch DR. The use of absorbable co-polymer pads with alginate and cells for articular cartilage repair in rabbits. Biomaterials 2003; 24: 2653-2660.
21. Cui Y, Wu J, Hu Y. Repair of articular cartilage defect with poly-lactide-co-glycolide loaded with recombinant human bone morphogenetic protein in rabbits. Chinese Journal of Reparative and Reconstructive Surgery 2007; 21: 1233-1237.
22. Cui YM, Wu J, Hu YY. Repairing articular cartilage defects in rabbits using bone marrow stromal cell-derived chondrocytes compounded with poly(lactic-co-glycolic acid). Journal of Clinical Rehabilitative Tissue Engineering Research 2009; 13: 10049-10054.
23. Dahlin RL, Kinard LA, Lam J, Needham CJ, Lu S, Kasper FK, et al. Articular chondrocytes and mesenchymal stem cells seeded on biodegradable scaffolds for the repair of cartilage in a rat osteochondral defect model. Biomaterials 2014; 35: 7460-7469.
24. De Girolamo L, Niada S, Arrigoni E, Di Giancamillo A, Domeneghini C, Dadsetan M, et al. Repair of osteochondral defects in the minipig model by OPF hydrogel loaded with adipose-derived mesenchymal stem cells. Regenerative Medicine 2015; 10: 135-151.
25. Deng T, Lv J, Pang J, Liu B, Ke J. Construction of tissue-engineered osteochondral composites and repair of large joint defects in rabbit. J Tissue Eng Regen Med 2014; 8: 546-556.
26. Di W, Zhao ZJ, Zhu XS, Hao Y, Qing L, Ke M. Calcium alginate beads combined with autologous chondrocyte for repair of adult rabbit articular cartilage injury. Journal of Clinical Rehabilitative Tissue Engineering Research 2010; 14: 3811-3814.
27. Diduch DR, Jordan LC, Mierisch CM, Balian G. Marrow stromal cells embedded in alginate for repair of osteochondral defects. Arthroscopy 2000; 16: 571-577.
28. Ding XP, Jin XQ, Wang ZY, Fu YL. Repairing articular cartilage defects in rabbits by gene-modified stem cells transplantation of biodegradable materials. Journal of Clinical Rehabilitative Tissue Engineering Research 2008; 12: 1839-1842.
29. Dorotka R, Bindreiter U, Macfelda K, Windberger U, Nehrer S. Marrow stimulation and chondrocyte transplantation using a collagen matrix for cartilage repair. Osteoarthritis Cartilage 2005; 13: 655-664.
30. Duan X, Zhu X, Dong X, Yang J, Huang F, Cen S, et al. Repair of large osteochondral defects in a beagle model with a novel type I collagen/glycosaminoglycan-porous titanium biphasic scaffold. Materials Science and Engineering: C 2013; 33: 3951-3957.

31. Duan P, Pan Z, Cao L, He Y, Wang H, Qu Z, et al. The effects of pore size in bilayered poly(lactide-co-glycolide) scaffolds on restoring osteochondral defects in rabbits. Journal of Biomedical Materials Research Part A 2014; 102: 180-192.
32. Endo J, Watanabe A, Sasho T, Yamaguchi S, Saito M, Akagi R, et al. Utility of T2 mapping and dGEMRIC for evaluation of cartilage repair after allograft chondrocyte implantation in a rabbit model. Osteoarthritis Cartilage 2015; 23: 280-288.
33. Filova E, Rampichova M, Handl M, Lytvynets A, Halouzka R, Usvald D, et al. Composite hyaluronate-type I collagen-fibrin scaffold in the therapy of osteochondral defects in miniature pigs. Physiological Research 2007; 56: S5-S16.
34. Filova E, Jelinek F, Handl M, Lytvynets A, Rampichova M, Varga F, et al. Novel composite hyaluronan/type I collagen/fibrin scaffold enhances repair of osteochondral defect in rabbit knee. Journal of Biomedical Materials Research Part B 2008; 87: 415-424.
35. Fonseca C, Caminal M, Peris D, Barrachina J, Fabregas PJ, Garcia F, et al. An arthroscopic approach for the treatment of osteochondral focal defects with cell-free and cell-loaded PLGA scaffolds in sheep. Cytotechnology 2014; 66: 345-354.
36. Fragonas E, Valente M, Pozzi-Mucelli M, Toffanin R, Rizzo R, Silvestri F, et al. Articular cartilage repair in rabbits by using suspensions of allogenic chondrocytes in alginate. Biomaterials 2000; 21: 795-801.
37. Frenkel SR, Toolan B, Menche D, Pitman MI, Pachence JM. Chondrocyte transplantation using a collagen bilayer matrix for cartilage repair. The Journal of Bone and Joint Surgery. British Volume 1997; 79: 831-836.
38. Frohbergh ME, Guevara JM, Grelsamer RP, Barbe MF, He X, Simonaro CM, et al. Acid ceramidase treatment enhances the outcome of autologous chondrocyte implantation in a rat osteochondral defect model. Osteoarthritis Cartilage 2016; 24: 752-762.
39. Gang J, Wei-yuan X, Ya Z, Xing-xiang Z, Fei Y, Wei-ping S. Repair of rabbit articular cartilage and subchondral defects using porous silk fibroin/hydroxyapatite combined with adipose-derived stromal cells. Journal of Clinical Rehabilitative Tissue Engineering Research 2011; 15: 5327-5333.
40. Gao J, Dennis JE, Solchaga LA, Goldberg VM, Caplan AI. Repair of osteochondral defect with tissue-engineered two-phase composite material of injectable calcium phosphate and hyaluronan sponge. Tissue Engineering Part A 2002; 8: 827-837.
41. Gao SJ, Wei JC, Lu B, Shao DC, Li T, Chen JQ, et al. Experimental research on repairing full-thickness articular cartilage defects by transplantation of autologous uncultured bone-marrow-derived mononuclear cells in combination with micro-fracture. Chinese Medical Journal 2012; 92: 2463-2467.
42. Getgood A, Henson F, Skelton C, Herrera E, Brooks R, Fortier LA, et al. The Augmentation of a Collagen/Glycosaminoglycan Biphasic Osteochondral Scaffold with Platelet-Rich Plasma and Concentrated Bone Marrow Aspirate for Osteochondral Defect Repair in Sheep: A Pilot Study. Cartilage 2012; 3: 351-363.
43. Gong L, Zhou X, Wu Y, Zhang Y, Wang C, Zhou H, et al. Proteomic analysis profile of engineered articular cartilage with chondrogenic differentiated adipose tissue-derived stem cells loaded polyglycolic acid mesh for weight-bearing area defect repair. Tissue Engineering Part A 2014; 20: 575-587.

44. Guenther D, Liu C, Horstmann H, Krettek C, Jagodzinski M, Haasper C. Near-infrared spectroscopy correlates with established histological scores in a miniature pig model of cartilage regeneration. The Open Orthopaedics Journal 2014; 8: 93-99.
45. Guo CA, Liu XG, Huo JZ, Jiang C, Wen XJ, Chen ZR. Novel gene-modified-tissue engineering of cartilage using stable transforming growth factor-beta1-transfected mesenchymal stem cells grown on chitosan scaffolds. Journal of Bioscience and Bioengineering 2007; 103: 547-556.
46. Guo X, Park H, Young S, Kretlow JD, van den Beucken JJ, Baggett LS, et al. Repair of osteochondral defects with biodegradable hydrogel composites encapsulating marrow mesenchymal stem cells in a rabbit model. Acta Biomaterialia 2010; 6: 39-47.
47. Gupta A, Bhat S, Jagdale PR, Chaudhari BP, Lidgren L, Gupta KC, et al. Evaluation of three-dimensional chitosan-agarose-gelatin cryogel scaffold for the repair of subchondral cartilage defects: an in vivo study in a rabbit model. Tissue Engineering Part A 2014; 20: 3101-3111.
48. Han NB, Zhao JN, Zhang Y, He J, Lu X, Wang JL, et al. Rabbit knee joint cartilage defect repaired by transforming growth factor-beta1-induced heparinized collagen/Chitosan scaffold binding with adipose-derived stem cells. Journal of Clinical Rehabilitative Tissue Engineering Research 2009; 13: 6611-6616.
49. Hao JL, Wang YL, Sun ZY, Xia YY, Shi ZL, Zhu LY, et al. Repairing articular cartilage full-thickness defects with homograft of mesenchymal stem cells seeded onto novel scaffold composites hydroxyapatite/calcium polyphosphate/poly-L-lactide. Chinese Journal of Clinical Rehabilitation 2006; 10: 64-67.
50. He DD, Zeng LY, Xiang C, Wang YZ, Wang SD, Duan WP, et al. Repairing articular cartilage defects in the knee of rabbits using type I/III-collagen- membrane combined with autologous bone marrow mesenchymal stem cells. Chinese Journal of Tissue Engineering Research 2012; 16: 7031-7036.
51. Ho ST, Hutmacher DW, Ekaputra AK, Hitendra D, Hui JH. The evaluation of a biphasic osteochondral implant coupled with an electrospun membrane in a large animal model. Tissue Engineering Part A 2010; 16: 1123-1141.
52. Igarashi T, Iwasaki N, Kasahara Y, Minami A. A cellular implantation system using an injectable ultra-purified alginate gel for repair of osteochondral defects in a rabbit model. Journal of Biomedical Materials Research Part A 2010; 94: 844-855.
53. Igarashi T, Iwasaki N, Kawamura D, Kasahara Y, Tsukuda Y, Ohzawa N, et al. Repair of articular cartilage defects with a novel injectable in situ forming material in a canine model. Journal of Biomedical Materials Research Part A 2012; 100: 180-187.
54. Im GI, Ahn JH, Kim SY, Choi BS, Lee SW. A hyaluronate-atelocollagen/beta-tricalcium phosphate-hydroxyapatite biphasic scaffold for the repair of osteochondral defects: a porcine study. Tissue Engineering Part A 2010; 16: 1189-1200.
55. Im GI, Lee JH. Repair of osteochondral defects with adipose stem cells and a dual growth factor-releasing scaffold in rabbits. Journal of Biomedical Materials Research Part B 2010; 92: 552-560.
56. Ito Y, Ochi M, Adachi N, Sugawara K, Yanada S, Ikada Y, et al. Repair of osteochondral defect with tissue-engineered chondral plug in a rabbit model. Arthroscopy 2005; 21: 1155-1163.
57. Ito Y, Adachi N, Nakamae A, Yanada S, Ochi M. Transplantation of tissue-engineered osteochondral plug using cultured chondrocytes and interconnected porous calcium hydroxyapatite ceramic cylindrical plugs to treat osteochondral defects in a rabbit model. Artificial Organs 2008; 32: 36-44.

58. Jagodzinski M, Liu C, Guenther D, Burssens A, Petri M, Abedian R, et al. Bone marrow-derived cell concentrates have limited effects on osteochondral reconstructions in the mini pig. Tissue Engineering Part C 2014; 20: 215-226.
59. Jang KM, Lee JH, Park CM, Song HR, Wang JH. Xenotransplantation of human mesenchymal stem cells for repair of osteochondral defects in rabbits using osteochondral biphasic composite constructs. Knee Surgery, Sports Traumatology, Arthroscopy 2014; 22: 1434-1444.
60. Jiang CC, Chiang H, Liao CJ, Lin YJ, Kuo TF, Shieh CS, et al. Repair of porcine articular cartilage defect with a biphasic osteochondral composite. Journal of Orthopaedic Research 2007; 25: 1277-1290.
61. Jurgens WJ, Kroeze RJ, Zandieh-Doulabi B, van Dijk A, Renders GA, Smit TH, et al. One-step surgical procedure for the treatment of osteochondral defects with adipose-derived stem cells in a caprine knee defect: a pilot study. BioResearch Open Access 2013; 2: 315-325.
62. Kandel RA, Grynpas M, Pilliar R, Lee J, Wang J, Waldman S, et al. Repair of osteochondral defects with biphasic cartilage-calcium polyphosphate constructs in a Sheep model. Biomaterials 2006; 27: 4120-4131.
63. Kang H, Peng J, Lu S, Liu S, Zhang L, Huang J, et al. In vivo cartilage repair using adipose-derived stem cell-loaded decellularized cartilage ECM scaffolds. Journal of Tissue Engineering and Regenerative Medicine 2012.
64. Kayakabe M, Tsutsumi S, Watanabe H, Kato Y, Takagishi K. Transplantation of autologous rabbit BM-derived mesenchymal stromal cells embedded in hyaluronic acid gel sponge into osteochondral defects of the knee. Cytotherapy 2006; 8: 343-353.
65. Kazemnejad S, Khanmohammadi M, Mobini S, Taghizadeh-Jahed M, Khanjani S, Arasteh S, et al. Comparative repair capacity of knee osteochondral defects using regenerated silk fiber scaffolds and fibrin glue with/without autologous chondrocytes during 36 weeks in rabbit model. Cell Tissue Res 2016; 364: 559-572.
66. Kon E, Delcogliano M, Filardo G, Fini M, Giavaresi G, Francioli S, et al. Orderly osteochondral regeneration in a sheep model using a novel nano-composite multilayered biomaterial. J Orthop Res 2010; 28: 116-124.
67. Kose GT, Korkusuz F, Ozkul A, Soysal Y, Ozdemir T, Yildiz C, et al. Tissue engineered cartilage on collagen and PHBV matrices. Biomaterials 2005; 26: 5187-5197.
68. Kuo CY, Chen CH, Hsiao CY, Chen JP. Incorporation of chitosan in biomimetic gelatin/chondroitin-6-sulfate/hyaluronan cryogel for cartilage tissue engineering. Carbohydr Polym 2015; 117: 722-730.
69. Lee JC, Lee SY, Min HJ, Han SA, Jang J, Lee S, et al. Synovium-derived mesenchymal stem cells encapsulated in a novel injectable gel can repair osteochondral defects in a rabbit model. Tissue Engineering Part A 2012; 18: 2173-2186.
70. Lee JC, Min HJ, Park HJ, Lee S, Seong SC, Lee MC. Synovial membrane-derived mesenchymal stem cells supported by platelet-rich plasma can repair osteochondral defects in a rabbit model. Arthroscopy 2013; 29: 1034-1046.
71. Lee JM, Kim JD, Oh EJ, Oh SH, Lee JH, Im GI. PD98059-impregnated functional plga scaffold for direct tissue engineering promotes chondrogenesis and prevents hypertrophy from mesenchymal stem cells. Tissue Engineering Part A 2014; 20: 982-991.
72. Li Q, Tang JC, Sun ZY, Wang SK, Li WZ. Repairing full-thickness articular cartilage defects with homograft of mesenchymal stem cells seeded onto cancellous demineralized bone matrix. Journal of Clinical Rehabilitative Tissue Engineering Research 2008; 12: 8943-8947.

73. Li G, Fu N, Xie J, Fu Y, Deng S, Cun X, et al. Poly(3-hydroxybutyrate-co-4-hydroxybutyrate) based electrospun 3D scaffolds for delivery of autogeneic chondrocytes and adipose-derived stem cells: Evaluation of cartilage defects in rabbit. Journal of Biomedical Nanotechnology 2015; 11: 105-116.
74. Li X, Li Y, Zuo Y, Qu D, Liu Y, Chen T, et al. Osteogenesis and chondrogenesis of biomimetic integrated porous PVA/gel/V-n-HA/pa6 scaffolds and BMSCs construct in repair of articular osteochondral defect. Journal of Biomedical Materials Research Part A 2015.
75. Lim CT, Ren X, Afizah MH, Tarigan-Panjaitan S, Yang Z, Wu Y, et al. Repair of osteochondral defects with rehydrated freeze-dried oligo[poly(ethylene glycol) fumarate] hydrogels seeded with bone marrow mesenchymal stem cells in a porcine model. Tissue Engineering Part A 2013; 19: 1852-1861.
76. Lin J, Wang R, Cheng L. Articular cartilage defects repaired with homograft of mesenchymal stem cells seeded onto medical collagen membrane of guided tissue regeneration. Chinese Journal of Reparative and Reconstructive Surgery 2006; 20: 1229-1234.
77. Liu Y, Shu XZ, Prestwich GD. Osteochondral defect repair with autologous bone marrow-derived mesenchymal stem cells in an injectable, in situ, cross-linked synthetic extracellular matrix. Tissue Engineering 2006; 12: 3405-3416.
78. Liu M, Xiang Z, Pei F, Huang F, Cen S, Zhong G, et al. Repairing defects of rabbit articular cartilage and subchondral bone with biphasic scaffold combined bone marrow stromal stem cells. Chinese Journal of Reparative and Reconstructive Surgery 2010; 24: 87-93.
79. Liu J, Nie H, Xu Z, Niu X, Guo S, Yin J, et al. The effect of 3D nanofibrous scaffolds on the chondrogenesis of induced pluripotent stem cells and their application in restoration of cartilage defects. PLoS One 2014; 9: e111566.
80. Liu PF, Guo L, Zhao DW, Zhang ZJ, Kang K, Zhu RP, et al. Study of human acellular amniotic membrane loading bone marrow mesenchymal stem cells in repair of articular cartilage defect in rabbits. Genetics and Molecular Research 2014; 13: 7992-8001.
81. Lohan A, Marzahn U, El Sayed K, Haisch A, Muller RD, Kohl B, et al. Osteochondral articular defect repair using auricle-derived autologous chondrocytes in a rabbit model. Annals of Anatomy 2014; 196: 317-326.
82. Loken S, Jakobsen RB, Aroen A, Heir S, Shahdadfar A, Brinchmann JE, et al. Bone marrow mesenchymal stem cells in a hyaluronan scaffold for treatment of an osteochondral defect in a rabbit model. Knee Surgery, Sports Traumatology, Arthroscopy 2008; 16: 896-903.
83. Lopiz-Morales Y, Abarrategi A, Ramos V, Moreno-Vicente C, Lopez-Duran L, Lopez-Lacomba JL, et al. In vivo comparison of the effects of rhBMP-2 and rhBMP-4 in osteochondral tissue regeneration. European Cells and Materials 2010; 20: 367-378.
84. Ma HB, Li YX, Wang ML. Adipose-derived stem cells transfected with adenovirus carrying bone morphogenetic protein 14 for repair of articular cartilage injury. [Chinese]. Chinese Journal of Tissue Engineering Research 2015; 19: 54-60.
85. Maciulaitis J, Deveikyte M, Rekstyte S, Bratchikov M, Darinskas A, Simbelyte A, et al. Preclinical study of SZ2080 material 3D microstructured scaffolds for cartilage tissue engineering made by femtosecond direct laser writing lithography. Biofabrication 2015; 7: 015015.
86. Marmotti A, Bruzzone M, Bonasia DE, Castoldi F, Rossi R, Piras L, et al. One-step osteochondral repair with cartilage fragments in a composite scaffold. Knee Surgery, Sports Traumatology, Arthroscopy 2012; 20: 2590-2601.

87. Marmotti A, Bruzzone M, Bonasia DE, Castoldi F, Von Degerfeld MM, Bignardi C, et al. Autologous cartilage fragments in a composite scaffold for one stage osteochondral repair in a goat model. European Cells and Materials 2013; 26: 15-32.
88. Masuoka K, Asazuma T, Hattori H, Yoshihara Y, Sato M, Matsumura K, et al. Tissue engineering of articular cartilage with autologous cultured adipose tissue-derived stromal cells using atelocollagen honeycomb-shaped scaffold with a membrane sealing in rabbits. Journal of Biomedical Materials Research Part B 2006; 79: 25-34.
89. Mazaki T, Shiozaki Y, Yamane K, Yoshida A, Nakamura M, Yoshida Y, et al. A novel, visible light-induced, rapidly cross-linkable gelatin scaffold for osteochondral tissue engineering. Scientific Reports 2014; 4: 4457.
90. Meppelink AM, Zhao X, Griffin DJ, Erali RA, Gill TJ, Bonassar LJ, et al. Hyaline Articular Matrix formed by Dynamic Self-Regenerating Cartilage and Hydrogels. Tissue Eng Part A 2016.
91. Miao ZN, Pan YH, Zhu JZ, Qian HG, Zhao JD. Constructing tissue engineered cartilage with silk fibroin compound bone marrow mesenchymal stem cells. Journal of Clinical Rehabilitative Tissue Engineering Research 2008; 12: 5243-5247.
92. Miller RE, Grodzinsky AJ, Vanderploeg EJ, Lee C, Ferris DJ, Barrett MF, et al. Effect of self-assembling peptide, chondrogenic factors, and bone marrow-derived stromal cells on osteochondral repair. Osteoarthritis Cartilage 2010; 18: 1608-1619.
93. Mrosek EH, Schagemann JC, Chung HW, Fitzsimmons JS, Yaszemski MJ, Mardones RM, et al. Porous tantalum and poly-epsilon-caprolactone biocomposites for osteochondral defect repair: preliminary studies in rabbits. Journal of Orthopaedic Research 2010; 28: 141-148.
94. Munirah S, Samsudin OC, Chen HC, Salmah SH, Aminuddin BS, Ruszymah BH. Articular cartilage restoration in load-bearing osteochondral defects by implantation of autologous chondrocyte-fibrin constructs: an experimental study in sheep. The Journal of Bone and Joint Surgery. British Volume 2007; 89: 1099-1109.
95. Necas A, Planka L, Srnec R, Crha M, Hlucilova J, Klima J, et al. Quality of newly formed cartilaginous tissue in defects of articular surface after transplantation of mesenchymal stem cells in a composite scaffold based on collagen I with chitosan micro- and nanofibres. Physiological Research 2010; 59: 605-614.
96. Niederauer GG, M AS, Leatherbury NC, Korvick DL, Harroff Jr HH, Ehler WC, et al. Evaluation of multiphase implants for repair of focal osteochondral defects in goats. Biomaterials 2000; 21: 2561-2574.
97. Noguchi T, Oka M, Fujino M, Neo M, Yamamuro T. Repair of osteochondral defects with grafts of cultured chondrocytes. Comparison of allografts and isografts. Clinical Orthopaedics and Related Research 1994: 251-258.
98. Oliveira JT, Gardel LS, Rada T, Martins L, Gomes ME, Reis RL. Injectable gellan gum hydrogels with autologous cells for the treatment of rabbit articular cartilage defects. Journal of Orthopaedic Research 2010; 28: 1193-1199.
99. Oshima Y, Watanabe N, Matsuda K, Takai S, Kawata M, Kubo T. Behavior of transplanted bone marrow-derived GFP mesenchymal cells in osteochondral defect as a simulation of autologous transplantation. Journal of Histochemistry & Cytochemistry 2005; 53: 207-216.
100. Oshima Y, Harwood FL, Coutts RD, Kubo T, Amiel D. Variation of mesenchymal cells in polylactic acid scaffold in an osteochondral repair model. Tissue Engineering Part C 2009; 15: 595-604.

101. Pan Z, Duan P, Liu X, Wang H, Cao L, He Y, et al. Effect of porosities of bilayered porous scaffolds on spontaneous osteochondral repair in cartilage tissue engineering. Regen Biomater 2015; 2: 9-19.
102. Pei M, He F, Boyce BM, Kish VL. Repair of full-thickness femoral condyle cartilage defects using allogeneic synovial cell-engineered tissue constructs. Osteoarthritis Cartilage 2009; 17: 714-722.
103. Perka C, Schultz O, Sittinger M, Zippel H. Chondrocyte transplantation in PGLA/polydioxanone fleece. Der Orthopäde 2000; 29: 112-119.
104. Perka C, Schultz O, Spitzer RS, Lindenhayn K. The influence of transforming growth factor beta1 on mesenchymal cell repair of full-thickness cartilage defects. vol. 52, 3 ed. United States: John Wiley & Sons Inc John Wiley and Sons Inc. (New York, NY, United States) 2000:543-552.
105. Pilliar RM, Kandel RA, Grynpas MD, Zalzal P, Hurtig M. Osteochondral defect repair using a novel tissue engineering approach: Sheep model study. Technology and Health Care 2007; 15: 47-56.
106. Qi H, Sun L, Chen L, Tao JF, Jiang J. Demineralized bone matrix compounded with biological fibrin glue for repairing rabbit cartilage defects. Journal of Clinical Rehabilitative Tissue Engineering Research 2010; 14: 4589-4593.
107. Qi Y, Du Y, Li W, Dai X, Zhao T, Yan W. Cartilage repair using mesenchymal stem cell (MSC) sheet and MSCs-loaded bilayer PLGA scaffold in a rabbit model. Knee Surg Sports Traumatol Arthrosc 2014; 22: 1424-1433.
108. Reyes R, Pec MK, Sanchez E, del Rosario C, Delgado A, Evora C. Comparative, osteochondral defect repair: stem cells versus chondrocytes versus bone morphogenetic protein-2, solely or in combination. European Cells and Materials 2013; 25: 351-365; discussion 365.
109. Rudert M, Wilms U, Hoberg M, Wirth CJ. Cell-based treatment of osteochondral defects in the rabbit knee with natural and synthetic matrices: cellular seeding determines the outcome. Archives of Orthopaedic and Trauma Surgery 2005; 125: 598-608.
110. Sancho-Tello M, Forriol F, Gastaldi P, Ruiz-Sauri A, De Llano JJM, Novella-Maestre E, et al. Time evolution of in vivo articular cartilage repair induced by bone marrow stimulation and scaffold implantation in rabbits. International Journal of Artificial Organs 2015; 38: 210-223.
111. Schaefer D, Martin I, Jundt G, Seidel J, Heberer M, Grodzinsky A, et al. Tissue-engineered composites for the repair of large osteochondral defects. Arthritis & Rheumatology 2002; 46: 2524-2534.
112. Schagemann JC, Erggelet C, Chung HW, Lahm A, Kurz H, Mrosek EH. Cell-laden and cell-free biopolymer hydrogel for the treatment of osteochondral defects in a sheep model. Tissue Engineering Part A 2009; 15: 75-82.
113. Schleicher I, Lips KS, Sommer U, Schappat I, Martin AP, Szalay G, et al. Biphasic scaffolds for repair of deep osteochondral defects in a sheep model. Journal of Surgical Research 2013; 183: 184-192.
114. Seo JP, Tanabe T, Tsuzuki N, Haneda S, Yamada K, Furuoka H, et al. Effects of bilayer gelatin/beta-tricalcium phosphate sponges loaded with mesenchymal stem cells, chondrocytes, bone morphogenetic protein-2, and platelet rich plasma on osteochondral defects of the talus in horses. Research in Veterinary Science 2013; 95: 1210-1216.
115. Seol D, Magnetta MJ, Ramakrishnan PS, Kurriger GL, Choe H, Jang K, et al. Biocompatibility and preclinical feasibility tests of a temperature-sensitive hydrogel for the purpose of surgical

wound pain control and cartilage repair. Journal of Biomedical Materials Research Part B 2013; 101: 1508-1515.

116. Shao X, Goh JC, Hutmacher DW, Lee EH, Zigang G. Repair of large articular osteochondral defects using hybrid scaffolds and bone marrow-derived mesenchymal stem cells in a rabbit model. Tissue Engineering 2006; 12: 1539-1551.

117. Shao XX, Hutmacher DW, Ho ST, Goh JC, Lee EH. Evaluation of a hybrid scaffold/cell construct in repair of high-load-bearing osteochondral defects in rabbits. Biomaterials 2006; 27: 1071-1080.

118. Shimomura K, Moriguchi Y, Ando W, Nansai R, Fujie H, Hart DA, et al. Osteochondral Repair Using a Scaffold-Free Tissue-Engineered Construct Derived from Synovial Mesenchymal Stem Cells and a Hydroxyapatite-Based Artificial Bone. Tissue Engineering Part A 2014.

119. Solchaga LA, Gao J, Dennis JE, Awadallah A, Lundberg M, Caplan AI, et al. Treatment of osteochondral defects with autologous bone marrow in a hyaluronan-based delivery vehicle. Tissue Engineering 2002; 8: 333-347.

120. Song ZM, Zhang SK, Liu Y, Ren Q, Fu CF. Transgeneic allogenic tissue-engineered cartilage in repairing the full thickness defects of knee in rabbits. Chinese Journal of Clinical Rehabilitation 2006; 10: 60-63.

121. Sosio C, Di Giancamillo A, Deponti D, Gervaso F, Scalera F, Melato M, et al. Osteochondral repair by a novel interconnecting collagen-hydroxyapatite substitute: a large-animal study. Tissue Engineering Part A 2015; 21: 704-715.

122. Tan WC, Zha ZG, Zhang JQ, Zheng LH, Liang YZ, Xia JS, et al. Animal-origin osteochondral scaffold combined with bone marrow mesenchymal stem cells/chondrocytes for repair of composite osteochondral defects in rabbit knee joints. Journal of Clinical Rehabilitative Tissue Engineering Research 2011; 15: 2265-2269.

123. Tanaka T, Komaki H, Chazono M, Fujii K. Use of a biphasic graft constructed with chondrocytes overlying a beta-tricalcium phosphate block in the treatment of rabbit osteochondral defects. Tissue Engineering 2005; 11: 331-339.

124. Tatebe M, Nakamura R, Kagami H, Okada K, Ueda M. Differentiation of transplanted mesenchymal stem cells in a large osteochondral defect in rabbit. Cytotherapy 2005; 7: 520-530.

125. Veronesi F, Cadossi M, Giavaresi G, Martini L, Setti S, Buda R, et al. Pulsed electromagnetic fields combined with a collagenous scaffold and bone marrow concentrate enhance osteochondral regeneration: an in vivo study. BMC Musculoskelet Disord 2015; 16: 233.

126. Wakitani S, Goto T, Pineda SJ, Young RG, Mansour JM, Caplan AI, et al. Mesenchymal cell-based repair of large, full-thickness defects of articular cartilage. The Journal of Bone and Joint Surgery 1994; 76: 579-592.

127. Wang G, Li D, Gu GS, Sun DH, Wang CX, Xu P. Influences of different inductive methods on cartilage repair by tissue engineered cartilage with rabbit mesenchymal stem cells. Journal of Jilin University Medicine Edition 2006; 32: 981-984.

128. Wang Y, Chen X, Wang LG. Bone marrow monocytes and porous lactic acid mixture in the repair of rabbit articular cartilage defects. Journal of Clinical Rehabilitative Tissue Engineering Research 2007; 11: 10128-10130.

129. Wang W, Li B, Li Y, Jiang Y, Ouyang H, Gao C. In vivo restoration of full-thickness cartilage defects by poly(lactide-co-glycolide) sponges filled with fibrin gel, bone marrow mesenchymal stem cells and DNA complexes. Biomaterials 2010; 31: 5953-5965.

130. Wang X, Li Y, Han R, He C, Wang G, Wang J, et al. Demineralized bone matrix combined bone marrow mesenchymal stem cells, bone morphogenetic protein-2 and transforming growth factor-beta3 gene promoted pig cartilage defect repair. PLoS One 2014; 9: e116061.
131. Wang Y, Li DH. Bone marrow mesenchymal stem cells combined with peptide hydrogel and chondrogenic factors for repair of articular cartilage defects in rabbits. [Chinese]. Chinese Journal of Tissue Engineering Research 2015; 19: 30-36.
132. Wang CC, Yang KC, Lin KH, Liu YL, Yang YT, Kuo TF, et al. Expandable Scaffold Improves Integration of Tissue-Engineered Cartilage: An In Vivo Study in a Rabbit Model. Tissue Eng Part A 2016; 22: 873-884.
133. Wayne JS, McDowell CL, Shields KJ, Tuan RS. In vivo response of polylactic acid-alginate scaffolds and bone marrow-derived cells for cartilage tissue engineering. Tissue Engineering 2005; 11: 953-963.
134. Wegener B, Schrimpf FM, Bergschmidt P, Pietschmann MF, Utzschneider S, Milz S, et al. Cartilage regeneration by bone marrow cells-seeded scaffolds. Journal of Biomedical Materials Research Part A 2010; 95: 735-740.
135. Wu J, Yang T, Liu Y, Guo T, Mu Y, Li Y. Preliminary studies on repairing osteochondral defects in the rabbit knee joint by using porous PA66/n-HA combination mesenchymal stem cells. Journal of Biomedical Engineering 2008; 25: 1349-1353.
136. Xie J, Han Z, Naito M, Maeyama A, Kim SH, Kim YH, et al. Articular cartilage tissue engineering based on a mechano-active scaffold made of poly(L-lactide-co-epsilon-caprolactone): In vivo performance in adult rabbits. Journal of Biomedical Materials Research Part B 2010; 94: 80-88.
137. Xie X, Wang Y, Zhao C, Guo S, Liu S, Jia W, et al. Comparative evaluation of MSCs from bone marrow and adipose tissue seeded in PRP-derived scaffold for cartilage regeneration. Biomaterials 2012; 33: 7008-7018.
138. Yan H, Yu C. Repair of full-thickness cartilage defects with cells of different origin in a rabbit model. Arthroscopy 2007; 23: 178-187.
139. Yang YJ, Zhu QS. Cartilage-derived morphogenetic protein growth factor 1-induced rat adipose-derived stem cells in repairing knee joint defect in rabbits. Academic Journal of Second Military Medical University 2009; 30: 1363-1366.
140. Yang Q, Peng J, Lu SB, Guo QY, Zhao B, Zhang L, et al. Evaluation of an extracellular matrix-derived acellular biphasic scaffold/cell construct in the repair of a large articular high-load-bearing osteochondral defect in a canine model. Chinese Medical Journal 2011; 124: 3930-3938.
141. Yin Z, Zhang L, Wang J. Repair of articular cartilage defects with "two-phase" tissue engineered cartilage constructed by autologous marrow mesenchymal stem cells and "two-phase" allogeneic bone matrix gelatin. Chinese Journal of Reparative and Reconstructive Surgery 2005; 19: 652-657.
142. Yin H, Sun Z, Sun X, Xu Y, Li P, Meng H, et al. Induction of mesenchymal stem cell chondrogenic differentiation and functional cartilage microtissue formation for in vivo cartilage regeneration by cartilage extracellular matrix-derived particles. Acta Biomaterialia 2016; 33: 96-109.
143. Zhao Q, Wang S, Tian J, Wang L, Dong S, Xia T, et al. Combination of bone marrow concentrate and PGA scaffolds enhance bone marrow stimulation in rabbit articular cartilage repair. Journal of Materials Science: Materials in Medicine 2013; 24: 793-801.

144. Zhao M, Chen Z, Liu K, Wan YQ, Li XD, Luo XW, et al. Repair of articular cartilage defects in rabbits through tissue-engineered cartilage constructed with chitosan hydrogel and chondrocytes. J Zhejiang Univ Sci B 2015; 16: 914-923.
145. Zhou XZ, Leung VY, Dong QR, Cheung KM, Chan D, Lu WW. Mesenchymal stem cell-based repair of articular cartilage with polyglycolic acid-hydroxyapatite biphasic scaffold. The International journal of Artificial Organs 2008; 31: 480-489.
146. Zhu W, Guo D, Peng L, Chen YF, Cui J, Xiong J, et al. Repair of rabbit cartilage defect based on the fusion of rabbit bone marrow stromal cells and Nano-HA/PLLA composite material. Artif Cells Nanomed Biotechnol 2016: 1-5.
