# Supplementary material for: Augmented cartilage regeneration by implantation of cellular versus acellular implants after bone marrow stimulation: a systematic review and meta-analysis of animal studies
Source: PeerJ. 2017 Oct 27;5:e3927. doi: 10.7717/peerj.3927 (PMC5661456; doi:10.7717/peerj.3927)

Supplementary Information 7. The forest plot illustrates the difference in cartilage regeneration between cellular and acellular biomaterials of each individual study.

	Experimental			Control				Mean Difference
Study or Subgroup	Mean	SD	Total	Mean	SD	Total	Weight	IV, Random, 95% CI
Yang et al 2011	27.3	14.72	16	55.71	12.58	8	1.0%	-28.41 [-39.72, -17.10]
Im et al 2010	64.72	11.5	9	75.61	11.5	9	1.0%	-10.89 [-21.52, -0.26]
Wang et al 2015	56	10.96	6	65.2	11.72	6	1.0%	-9.20 [-22.04, 3.64]
Miller et al 2010	50	10.61	7	58.21	10.49	6	1.0%	-8.21 [-19.71, 3.29]
Guenther et al 2014	56	8.14	5	63.68	16.89	5	0.9%	-7.68 [-24.11, 8.75]
Kon et al 2010	74.64	12.14	8	81.79	8.93	8	1.0%	-7.15 [-17.59, 3.29]
Christensen et al 2012	47.88	9.88	20	54.92	4.17	10	1.1%	-7.04 [-12.08, -2.00]
Caminal et al 2015	52.74	14.36	13	58.33	19.44	2	0.6%	-5.59 [-33.64, 22.46]
Lohan et al 2014	55.42	17.59	12	59.4	9.43	6	1.0%	-3.98 [-16.47, 8.51]
Filova et al 2007	66.67	11.02	6	70.14	5.89	5	1.0%	-3.47 [-13.69, 6.75]
Fonseca et al 2014	61.43	21.52	3	64.55	14.51	4	0.6%	-3.12 [-31.32, 25.08]
Liu et al 2014	38.73	4.36	12	41.68	3.43	12	1.2%	-2.95 [-6.09, 0.19]
Guo et al 2010	63.73	8.87	24	66.06	8.89	12	1.1%	-2.33 [-8.49, 3.83]
Wegener et al 2010	48.33	21.33	6	50	8.6	6	0.9%	-1.67 [-20.07, 16.73]
Jagodzinski et al 2014	47.22	12.22	5	47.78	5	5	1.0%	-0.56 [-12.13, 11.01]
Im et al 2010 (2)	48.39	11.51	6	48.34	12.76	6	1.0%	0.05 [-13.70, 13.80]
Ahn et al 2009	63.06	14.88	7	62.29	18.4	7	0.9%	0.77 [-16.76, 18.30]
Jurgens et al 2013	57.08	19.28	23	56.3	19.92	9	0.9%	0.78 [-14.43, 15.99]
Solchaga et al 2002	19.52	3.05	27	18.2	2.62	27	1.2%	1.32 [-0.20, 2.84]
Lee et al 2014	63.94	18.95	12	62.33	5.08	4	1.0%	1.61 [-10.21, 13.43]
de Girolamo et al 2015	49.49	2.74	7	47.21	2.17	7	1.2%	2.28 [-0.31, 4.87]
Niederauer et al 2000	48.71	14.2	40	46.43	14.08	24	1.1%	2.28 [-4.87, 9.43]
Gupta et al 2015	49.87	25.4	15	47.57	24.03	15	0.9%	2.30 [-15.39, 19.99]
Getgood et al 2012	45.2	7.39	12	41.74	7.31	12	1.1%	3.46 [-2.42, 9.34]
Ho et al 2010	53.69	19.64	24	49.48	14.67	12	1.0%	4.21 [-7.22, 15.64]
Chang et al 2011	52.95	21.87	16	47.86	21.43	8	0.9%	5.09 [-13.22, 23.40]
Li et al 2015	59.38	8.75	20	52.19	7.06	20	1.1%	7.19 [2.26, 12.12]
Igarashi et al 2012	73.57	7.5	8	66.07	5	8	1.1%	7.50 [1.25, 13.75]
Betsch et al 2013	71.44	10.32	14	62.73	16.25	14	1.0%	8.71 [-1.37, 18.79]
Lee et al 2012	59.23	25.78	12	50	23.36	12	0.8%	9.23 [-10.45, 28.91]
Yin et al 2005	92.86	4.57	18	82.85	4.84	18	1.2%	10.01 [6.93, 13.09]
Köse et al 2005	52.69	24.95	16	42.09	7.75	16	1.0%	10.60 [-2.20, 23.40]
Endo et al 2015	45.72	17.03	15	34.76	18.25	15	1.0%	10.96 [-1.67, 23.59]
Zhao et al 2015	70.81	17.81	9	59.58	22.98	9	0.8%	11.23 [-7.76, 30.22]
Wayne et al 2005	47.95	8	20	36.58	9.95	20	1.1%	11.37 [5.77, 16.97]
Bal et al 2010	44.17	4.86	30	32.23	8.37	30	1.2%	11.94 [8.48, 15.40]
Betsch et al 2014	70.52	15.18	14	58.02	14.61	14	1.0%	12.50 [1.46, 23.54]
Perka et al 2000	54.29	12.1	28	41.79	12.33	14	1.1%	12.50 [4.64, 20.36]
Kayakabe et al 2006	53.72	20.25	38	41.19	18.03	9	1.0%	12.53 [-0.89, 25.95]
Qi et al 2010	47.19	18.54	15	33.81	20.01	15	1.0%	13.38 [-0.42, 27.18]
Chung et al 2014	54.08	15.44	20	40.62	7.63	20	1.1%	13.46 [5.91, 21.01]
Masuoka et al 2006	63.58	6.57	12	48.93	4.94	12	1.1%	14.65 [10.00, 19.30]
Pan et al 2015	48.46	12.55	36	33.43	11.97	36	1.1%	15.03 [9.36, 20.70]
Qi et al 2014	53.75	18.84	24	38.34	15.22	12	1.0%	15.41 [3.97, 26.85]
Wang et al 2014	50.91	10.7	12	35.46	5.83	6	1.1%	15.45 [7.81, 23.09]
Xie et al 2012	64.41	11.14	24	48.04	5.75	12	1.1%	16.37 [10.85, 21.89]
Maciulaitis et al 2015	55.22	11.86	8	38.63	8.59	7	1.0%	16.59 [6.20, 26.98]
Duan et al 2014	55.54	14.16	58	38.91	13.16	58	1.1%	16.63 [11.66, 21.60]
Wang et al 2007	53.11	10.09	15	36.45	9.78	13	1.1%	16.66 [9.29, 24.03]
Chang et al 2013	70.79	15.9	16	53.24	13.8	12	1.0%	17.55 [6.52, 28.58]
Kang et al 2014	69.11	10.95	12	50.58	10.72	12	1.1%	18.53 [9.86, 27.20]
Ding et al 2008	44.32	20.95	22	25.58	12.03	11	1.0%	18.74 [7.46, 30.02]
Loken et al 2008	56.2	12.2	189	37.3	10.8	11	1.1%	18.90 [12.28, 25.52]
Kazemnejad et al 2016	58.83	10.47	12	39.71	3.89	12	1.1%	19.12 [12.80, 25.44]
Schagemann et al 2009	75.74	13.05	24	56.21	12.42	12	1.1%	19.53 [10.78, 28.28]
Mazaki et al 2014	50.58	16.9	12	30.14	14.66	12	1.0%	20.44 [7.78, 33.10]
Marmotti et al 2012	61.87	27.99	14	41.22	13.58	14	0.9%	20.65 [4.35, 36.95]
Frohberg et al 2016	66.5	17.91	12	45.5	4.36	6	1.0%	21.00 [10.28, 31.72]
Lim et al 2013	86.93	5.93	6	65.5	17.71	6	0.9%	21.43 [6.49, 36.37]
Zhao et al 2013	66.2	9.31	8	44.33	5.67	4	1.1%	21.87 [13.36, 30.38]
Xie et al 2010	60.69	18.82	8	37.07	9.82	8	0.9%	23.62 [8.91, 38.33]
Barron et al 2015	43.04	16.13	6	18.91	7.61	6	1.0%	24.13 [9.86, 38.40]
Di et al 2014	91.07	3.71	5	66.64	4.07	5	1.1%	24.43 [19.60, 29.26]
Jang et al 2014	43.89	30.23	12	19.03	8.07	12	0.9%	24.86 [7.16, 42.56]
Seo et al 2013	61.61	5.36	6	36.18	5.93	6	1.1%	25.43 [19.03, 31.83]
Veronesi et al 2015	87.3	10.08	10	61.11	10.2	10	1.1%	26.19 [17.30, 35.08]
Arakaki et al 2015	39.6	34.29	12	13.35	10.24	12	0.8%	26.25 [6.00, 46.50]
Song et al 2006	62.14	15.12	32	35.54	16.1	16	1.1%	26.60 [17.13, 36.07]
Dorotka et al 2005	53.69	11.94	7	24.17	5.39	6	1.1%	29.52 [19.68, 39.36]
Schaefer et al 2002	62.6	12.29	19	32.93	15.06	12	1.0%	29.67 [19.51, 39.83]
Marmotti et al 2013	77.91	8.09	4	47.61	5.15	4	1.1%	30.30 [20.90, 39.70]
Lin et al 2006	76.34	17.64	24	44.93	11.65	12	1.1%	31.41 [21.75, 41.07]
Yin et al 2016	83.79	9.7	24	51.93	6.85	24	1.1%	31.86 [27.11, 36.61]
Zhu et al 2016	72.5	14.61	12	40.36	10.95	12	1.0%	32.14 [21.81, 42.47]
Ito et al 2008	68.58	6.65	12	36.07	12.04	12	1.1%	32.51 [24.73, 40.29]
Shao et al 2006	62.43	19.88	9	29.92	25.25	6	0.7%	32.51 [8.49, 56.53]
Deng et al 2014	84.93	5.23	12	52.32	10.12	12	1.1%	32.61 [26.16, 39.06]
Frenkel et al 1997	74.43	9.29	23	41.09	16.14	23	1.1%	33.34 [25.73, 40.95]
Jiang et al 2007	60.5	3.44	20	26.5	3.14	18	1.2%	34.00 [31.91, 36.09]
Reyes et al 2013	81.13	13.55	24	46.99	27.4	12	0.9%	34.14 [17.72, 50.56]
Shimomura et al 2014	80.7	21.74	18	46.12	22.27	13	0.9%	34.58 [18.85, 50.31]
Ito et al 2005	70.36	7.04	12	34.29	12.55	12	1.1%	36.07 [27.93, 44.21]
Wang et al 2016	72.46	12.73	18	36.23	9.01	18	1.1%	36.23 [29.03, 43.43]
Han et al 2009	63.57	15.71	30	26.43	17.86	15	1.0%	37.14 [26.50, 47.78]
Ma et al 2015	44.29	14.35	16	7.14	4.79	8	1.1%	37.15 [29.37, 44.93]
Cao et al 2012	62.75	17.67	12	23.8	13.17	12	1.0%	38.95 [26.48, 51.42]
Necas et al 2014	61.25	15.92	10	22.08	12	10	1.0%	39.17 [26.81, 51.53]
Pei et al 2009	60.71	9.86	3	20.43	12	3	0.9%	40.28 [22.71, 57.85]
Zhou et al 2008	84.39	9.44	20	42.23	8.98	20	1.1%	42.16 [36.45, 47.87]
Gao et al 2012	82.15	13.09	10	39.29	20.29	10	0.9%	42.86 [27.89, 57.83]
Wang et al 2006	70.93	10.94	40	27.54	13.79	20	1.1%	43.39 [36.46, 50.32]
Wang et al 2010	64.15	21.98	24	20.29	6.21	9	1.1%	43.86 [34.18, 53.54]
Gong et al 2014	74.03	16.36	11	27.11	22.85	11	0.9%	46.92 [30.31, 63.53]
Cheng et al 2014	76.83	16.87	12	29.08	18.87	6	0.9%	47.75 [29.89, 65.61]
Bai et al 2008	83.47	12.38	8	34.36	10.89	8	1.0%	49.11 [37.68, 60.54]
Yan et al 2007	56.76	29.62	56	6.25	8.03	8	1.1%	50.51 [40.96, 60.06]
Duan et al 2013	79.13	5.13	8	27	6.06	8	1.1%	52.13 [46.63, 57.63]
Guo et al 2007	66.67	25.14	10	12.5	6.83	5	0.9%	54.17 [37.48, 70.86]
								**18.35 [15.08, 21.62]**

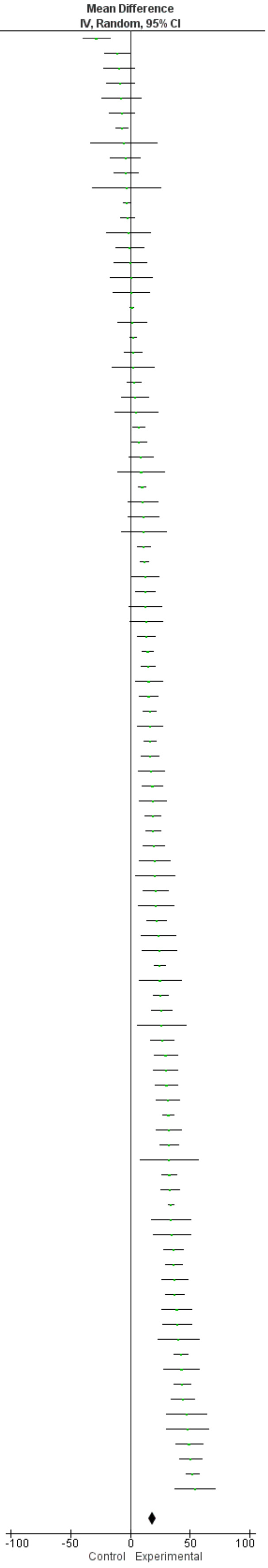

Supplement: Supplemental Information 9 [file peerj-05-3927-s009.pdf]
